# Impact of ejaculatory abstinence period and semen characteristic on the reproductive outcomes after intracytoplasmic sperm injection

**DOI:** 10.5935/1518-0557.20210124

**Published:** 2022

**Authors:** Elham Azizi, Mohammad Naji, Saghar Salehpour, Nasrin Saharkhiz, Maryam Karimi, Nasrin Borumandnia, Zahra Shams Mofarahe

**Affiliations:** 1 Department of Biology and Anatomical Sciences, School of Medicine, Shahid Beheshti University of Medical Sciences, Tehran, Iran; 2 Urology and Nephrology Research Center (UNRC), Shahid Beheshti University of Medical Sciences, Tehran, Iran; 3 IVF center, Taleghani Hospital, Shahid Beheshti University of Medical Sciences, Tehran, Iran; 4 Preventative Gynecology Research Center (PGRC), Imam Hossein Hospital, Shahid Beheshti University of Medical Sciences, Tehran, Iran; 5 Taleghani Hospital Research Development Unit, School of Medicine, Shahid Beheshti University of Medical Sciences, Tehran, Iran

**Keywords:** ICSI, fertilization, pregnancy, sexual abstinence, semen

## Abstract

**Objective:**

The prognostic of semen characteristics in intracytoplasmic sperm injection (ICSI) outcomes is not clear. Also, there is no evidence-based recommendation for the abstinence period before ICSI. So, we aimed to assess the influence of the abstinence period and semen characteristics on ICSI outcomes.

**Methods:**

A total of 1003 fresh ICSI cycles were divided into six groups; group 1 (1-day abstinence), group 2 (2 days abstinence), group 3 (3 days abstinence), group 4 (4 days abstinence), group 5 (5 days abstinence), and group 6 (6-10 days abstinence).

**Results:**

We showed that semen volume (p=0.0001) and total sperm count (*p*=0.005) were increased in the groups with higher abstinence periods. Other semen parameters did not significantly associate with the abstinence period. The percentage of progressively motile sperm was associated with fertilization rate (*p*=0.007), and the sperm morphology was associated with cleavage-stage embryo rate (*p*=0.036). No influence of abstinence or semen parameters on rates of pregnancies was observed.

**Conclusions:**

The abstinence period before ICSI can influence the semen volume and total sperm count, and possibly fertilization. Although the sperm with the highest quality are selected for ICSI, the percentages of progressively motile and morphologically normal sperm in the ejaculated semen have a predictive value for fertilization and cleavage rates after ICSI, respectively.

## INTRODUCTION

Spermatogenesis takes place in the seminiferous tubules of the testis, and then the immature spermatozoa migrate to the epididymis, where they develop their final state of maturity in the male reproductive tract and are stored in cauda epididymis until ejaculation (Rowley *et al.*, 1970; Amann & Howards, 1980; Johnson, 1982; Johnson & Varner, 1988; Robaire *et al.*, 2006). The sexual abstinence period, duration of the mature sperm storage in the epididymis can influence semen parameters. Longer sexual abstinence leads to increased semen volume and sperm count (Raziel *et al.*, 2001; De Jonge *et al.*, 2004; Jurema *et al.*, 2005; Sobreiro *et al.*, 2005; Marshburn *et al.*, 2010; Sunanda *et al.*, 2014; Agarwal *et al.*, 2016; Welliver *et al.*, 2016; Hanson *et al.*, 2018). However, the influence of the abstinence period on other semen quality parameters such as sperm motility, morphology, viability, and DNA integrity is still inconclusive and controversial (Hanson *et al.*, 2018). Although the abstinence period may affect some semen parameters, the optimal abstinence period for assisted reproductive technology (ART) cycles has remained to be elucidated.

Currently, most ART clinics use the World Health Organization (WHO) recommendation for a sexual abstinence period (2-7 days) before ART cycles. WHO recommended this period for the evaluation of semen quality in the primary assessment of male fertility (WHO, 2010). In this regard, the European Society of Human Reproduction and Embryology (ESHRE) has suggested a shorter period of sexual abstinence (3-4 days) for semen quality evaluation (Barratt *et al.*, 2011). Nevertheless, the WHO recommendation on the sexual abstinence period has been challenged by some studies that reported superior semen quality in different abstinence days from the recommended period (De Jonge *et al.*, 2004; Levitas *et al.*, 2005; Mayorga-Torres *et al.*, 2015; Agarwal *et al.*, 2016).

The optimal abstinence period may improve some functional semen parameters, with substantial roles in fertilization potential, which cannot be appraised in routine semen quality evaluation. Hence, it is necessary to determine the optimal abstinence period in which human spermatozoa have the highest fertilization potential for improvement of ART success rate. Concerning the optimal sexual abstinence period for ART procedures, there are a few studies with contradictory findings. Some studies have reported that a shorter abstinence period can improve pregnancy outcomes in intracytoplasmic sperm injection (ICSI) cycles (Colturato *et al.*, 2007; Periyasamy *et al.*, 2017; Borges Jr *et al.*, 2019), but another group has reported no correlation between abstinence period and ICSI outcomes (Lee *et al.*, 2015; 2018). Lee *et al*. (2018) have even indicated that the abstinence period beyond the recommended period of WHO did not decline pregnancy outcomes. Furthermore, semen samples collected after recurrent ejaculations and final abstinence of 12 hours could improve pregnancy outcomes in ICSI cycles (Sánchez-Martín *et al.*, 2013). The highest pregnancy outcomes with IUI were observed following less than three days of abstinence (Sugiyam *et al.*, 2008)2008. These findings have questioned the clinical application of WHO and ESHRE recommendations for the duration of the sexual abstinence period in ART (Ayad *et al.*, 2018). Therefore, defining the optimal sexual abstinence period for clinical application in ART should be considered by WHO and ESHRE.

ICSI procedure is the most widely used technique in ART, which may utilize for male or non-male factor infertility (European IVF-monitoring Consortium, 2017). Although in this procedure, only one sperm is selected by the embryologist for the injection into the cytoplasm of the oocyte, the semen quality may have prognostic value for the success of the ICSI. However, the literature regarding the prognostic value of the semen parameters, which may be affected by the abstinence period, on the ICSI outcomes are scanty, and the association is still under debate. However, some recent studies have been reported the semen quality might influence the outcomes of ICSI cycles (Chapuis *et al.*, 2017; Mazzilli *et al.*, 2017; Bartolacci *et al.*, 2018).

The influence of the abstinence period and also semen quality on the subsequent ICSI cycle outcomes is a conflicting issue in the literature (Borges Jr *et al.*, 2019; Setti *et al.*, 2020). Therefore, in this study, we evaluated the association between the sexual abstinence period and semen quality, fertilization rate, cleavage-stage embryo rate, and pregnancy outcomes after fresh ICSI cycles.

## MATERIAL AND METHODS

This retrospective study has assessed 1003 patients who underwent ICSI cycles, from December 2014 to August 2019. Fresh embryo transferred (ET) ICSI cycles were only included in this study. ICSI cycles that used sperm from testicular biopsies, males with hepatitis, pre-implantation genetic diagnosis, donation or activation of oocytes, frozen sperm, and oocyte *in vitro* maturation (IVM) were excluded. All procedures were performed according to guidelines of the ethics committee and informed consent was waived for the current study (IR.SBMU.RETECH.REC.1398.201).

### Semen collection and sperm preparation

The sexual abstinence period was asked before collecting semen samples. Semen samples were collected in our laboratory at the time of oocyte retrieval by masturbation into a wide-mouth specimen container. Samples were liquefied at 37°C for 30-60 min, before evaluation. Analysis of neat semen was performed for volume, sperm count, motility, and morphology according to the WHO criteria (WHO, 2010). Sperm concentration was the number of spermatozoa/mL in the semen. The total sperm count was determined by multiplying the sperm concentration by the volume of ejaculated semen. The percentage of motile sperm was evaluated among 200 spermatozoa in the semen, and the total sperm motility was the sum of the number of progressive and non-progressive spermatozoa. The percentage of the morphologically normal sperm was assessed using the Diff-quick method (Diff-Quick, Quick-Panoptic, Amposta, Spain). The swim-up method was used for the preparation of sperm before the ICSI procedure.

### Controlled ovarian stimulation, oocyte retrieval, and ICSI

Ovarian stimulation was performed either with GnRH antagonist or agonist protocols, based on specific parameters of the patients such as female age, body mass index (BMI), ovarian reserve, and medical history. Stimulation in patients was initiated with recombinant FSH (Gonal-F, Merck Serono Europe Ltd, UK). When at least 3 follicles >17 mm diameter were visualized by ultrasonography, human chorionic gonadotropin (hCG) (Pregnyl; MSD, Brussels, Belgium) trigger was administrated. 34-38 hours after hCG administration, cumulus-oocyte complexes were retrieved from both ovaries, by transvaginal aspiration under ultrasound guidance. After denudation of retrieved oocytes, and removal of surrounding cumulus cells, all matured oocytes were inseminated.

### Embryo transfer and luteal phase support

The embryos were cultured in a Global medium (Life Global^®^ ART media) and incubated in 6% CO_2_ at 37°C. Between 18-20 hours after insemination, fertilization was monitored by the presence of two pronuclei and two clear polar bodies in perivitelline space. Embryos were assessed and graded in cleavage-stage according to the number, size, and symmetry of blastomeres and the degree of fragmentation (Puissant *et al.*, 1987). For embryo transfer, 1-3 embryos were transferred to the uterine either on day 2 or 3. Biochemical pregnancy was determined by measuring beta-hCG levels in the blood sample test on day 14 after oocyte retrieval. Clinical pregnancy was determined by visualization of a gestational sac in ultrasound at weeks 5-6.

### Data analysis

Based on the sexual abstinence period, the cycles were divided into six groups (1, 2, 3, 4, 5, and 6-10 days of abstinence). The main outcome measures of this study were the rates of fertilization, cleavage-stage embryos, biochemical and clinical pregnancies.

Statistical analysis was carried out using SPSS software 22 (SPSS Inc., Chicago, IL). Data were presented as mean±standard deviation for continuous variables and frequency (percentage) for categorical variables. Kruskal-Wallis (with Dunn’s multiple comparisons test) and χ^2^ test were utilized for analysis of continuous and categorical variables, respectively.

In the multivariable analysis, we assessed the effect of abstinence period and also semen parameters (semen volume, total sperm count, sperm concentration, and the percentage of total motile, progressively motile, and morphologically normal sperm) on the main outcome measures. Female age, male age, early or secondary fertility, previous cycles, stimulation, duration of infertility, number of aspirated oocytes, number of transferred embryos, etiology (combined *vs*. Other, male factor *vs*. Other, unexplained *vs*. Other), ovarian stimulation protocol (antagonist *vs*. agonist) were also considered as potential confounding variables. To adjust the effects of abstinence period, semen parameters, and other confounding variables, multivariate logistic regression, and multiple linear regression were performed for dichotomous (biochemical pregnancy and clinical pregnancy), and continuous outcomes (fertilization and cleavage-stage embryo rate), respectively. A *p*-value of <0.05 was considered statistically significant.

## RESULTS

A total of 1003 fresh ICSI cycles were analyzed in this study and categorized into six groups based on days of abstinence period; group 1 (1 day, n=37), group 2 (2 days, n=170), group 3 (3 days, n=392), group 4 (4 days, n=253), group 5 (5 days, n=102), and group 6 (6-10 days, n=49). From 1003 studied patients, 21 patients had more than 7 days of abstinence period, which were more than WHO recommendation. No significant differences were detected in baseline characteristics of subjects in different studied groups, except for female age differences between groups 2 *vs*. 4 (*p*=0.01) (Table 1).

**Table 1:** Demographic and cycle characteristics of patients undergoing ICSI.

Parameter	Abstinence period						*p*-value
	Group 1^a^ (n=37)	Group 2^a^ (n=170)	Group 3^a^ (n=392)	Group 4^a^ (n=253)	Group 5^a^ (n=102)	Group 6^a^ (n=49)	
**Female age (y)**	33±6.1	34±5.9^b^	33±6.0	32±5.9^b^	34±5.6	32±5.1	0.01
**Male age (y)**	37±5.1	38±6.3	37±6.4	36±5.4	37±6.7	36±5.9	0.1
**Type of infertility** Primary n (%) Secondary n (%)	30 (81.1) 7 (18.9)	131 (77.5) 38 (22.5)	328 (83.7) 64 (16.3)	201 (79.5) 52 (20.5)	81 (79.4) 21 (20.6)	44 (89.8) 5 (10.2)	0.2
**Infertility duration (y)**	6.7±7.1	6.2±5.6	5.4±4.2	4.7±3.7	5.3±4.2	4.5±4.0	0.1
**No. of previous ICSI cycles N (%)** 0 1 2 3 & more	27 (72.9) 5 (13.5) 3 (8.1) 2 (5.4)	114 (68.7) 31 (18.7) 17 (10.2) 4 (2.4)	269 (68.6) 74 (18.8) 33 (8.5) 16 (4.1)	170 (67.2) 56 (22.1) 21 (8.3) 6 (2.4)	70 (68.6) 22 (21.6) 6 (5.9) 4 (3.9)	36 (73.5) 9 (18.4) 3 (6.1) 1 (2)	0.9
**Etiology N (%)** Male infertility PCOS Endometriosis Tubal factor Uterine Endocrinologydisorder Unexplained Combined	7 (18.9) 1 (2.8) 0 (0) 0 (0) 0 (0) 0 (0) 3 (8.1) 26 (70.2)	20 (11.8) 6 (3.5) 0 (0) 2 (1.2) 8 (4.7) 7 (4.1) 9 (5.3) 118 (69.4)	82 (20.9) 18 (4.6) 1 (0.3) 16 (4.1) 3 (0.7) 15 (3.8) 24 (6.1) 234 (59.5)	46 (18.2) 10 (4) 0 (0) 6 (2.4) 1 (0.4) 5 (2) 7 (2.7) 178 (70.3)	26 (25.5) 5 (4.9) 0 (0) 5 (4.9) 2 (2) 3 (2.9) 4 (3.9) 57 (55.9)	5 (10.2) 1 (2.1) 0 (0) 1 (2) 1 (2) 1 (2) 3 (6.1) 37 (75.6)	0.06
**Stimulation protocol** Antagonist N (%) Agonist N (%)	37 (100) 0 (0)	161 (94.7) 9 (5.3)	361 (92.1) 31 (7.9)	238 (94.07) 15 (5.92)	238 (94.07) 15 (5.92)	47 (95.9) 2 (4.1)	0.2

Data were presented as mean ± SD or frequency (percentage).

a The ICSI cycles were divided into six groups based on days of abstinence period: Group 1: 1 day abstinence, Group 2: 2 days abstinence, Group 3: 3 days abstinence, Group 4: 4 days abstinence, Group 5: 5 days abstinence, Group 6: 6-10 days abstinence

b Values with the same superscript in a row were significantly different.

Among ICSI cycle variables, there were no significant differences in the number of aspirated, injected, and fertilized oocytes, and the number of transferred embryos. In studied semen parameters, semen volume and also total sperm count were significantly increased in the groups with higher abstinence periods (*p*=0.0001 and *p*=0.005, respectively). Sperm concentration, the percentage of total motile, progressively motile, and morphologically normal sperm did not significantly differ among studied groups (Table 2). The highest rate of biochemical (32.4%) and clinical (31.1%) pregnancy were noticed in group 5 (5 days of abstinence) but no significant differences were observed among studied groups. There were also no significant differences in fertilization and cleavage-stage embryo rates of the studied groups (Table 2).

**Table 2:** Characteristics and outcomes of ICSI cycles.

Parameter	Abstinence period						*p*-value
	Group 1^a^ (n=37)	Group 2^a^ (n=170)	Group 3^a^ (n=392)	Group 4^a^ (n=253)	Group 5^a^ (n=102)	Group 6^a^ (n=49)	
**Semen volume (mL)**	2.6±1.4^c^	2.9±1.5^b^	3.4±1.5^cb^	3.4±1.5c^b^	3.6±1.5^cb^	3.6±1.7^c^	<0.0001
**Total sperm count**	118±126^c^	139±117^b^	160±134	157±115	196±127	213±158^cb^	0.005
**Sperm concentration**	46±41	52±37	49±30	50±32	49±30	61±36	0.1
**Total sperm motility (%)**	51±18	51±20	54±19	51±20	48±20	54±19	0.05
**Progressive motility of sperm (%)**	41±21	41±19	43±19	42±19	40±20	44±20	0.6
**Sperm morphology (%)**	1.8±2.3	2.1±2.0	2.3±2.0	2.2±1.9	2.6±2.2	2.2±2.0	0.2
**No. oocytes aspirated**	8.8±6.5	8.2±6.2	8.4±5.9	7.8±6.1	8.3±5.8	8.6±6.3	0.5
**No. oocytes injected**	6.8±5.3	6.4±4.8	6.6±4.9	6.3±5.1	6.4±4.1	6.9±5.4	0.8
**No. oocytes fertilized**	4.4±3.7	4.6±3.8	4.7±3.7	4.3±4.1	4.6±3.3	5.0±4.6	0.4
**No. embryos transferred**	1.6±0.5	1.9±0.4	1.8±0.5	1.9±0.4	1.9±0.5	1.7±0.5	0.03
**Fertilization rate (%)**	66±32	72±30	73±27	68±32	67±29	69±32	0.4
**Cleavage-stage embryo rate (%)**	89±29	91±25	92±22	91±23	92±24	93±16	0.8
**Biochemical pregnancy rate per embryo transfer N (%)**	8 (29.1)	35 (29.4)	90 (30.2)	58 (30.0)	24 (32.4)	11 (27.8)	0.9
**Clinical pregnancy rate per embryo transfer N (%)**	8 (29.2)	30 (25.2)	84 (28.1)	52 (26.9)	23 (31.1)	11 (27.8)	0.9

Data were presented as mean ± SD or frequency (percentage).

a The ICSI cycles were divided into six groups based on days of abstinence period: Group 1: 1 day abstinence, Group 2: 2 days abstinence, Group 3: 3 days abstinence, Group 4: 4 days abstinence, Group 5: 5 days abstinence, Group 6: 6-10 days abstinence

b,c Values with the same superscript in a row were significantly different.

A multivariable analysis was carried out to adjust the outcome measures of the study for potentially confounding variables (Table 3). It was revealed that the progressive motility of sperm was associated with fertilization rate (B=1.48, CI=0.40, 2.55, *p*=0.007) (Table 3). Moreover, sperm morphology was associated with cleavage-stage embryo rate (B=-0.64, CI=-1.23,-0.041, *p*=0.036) (Table 3). There is not a significant association between the abstinence period and the fertilization rate (B=-1.42, CI=-3.05, 0.20, *p*=0.087) (Table 3). Nevertheless, there was no association between abstinence or semen parameters and rates of pregnancies.

**Table 3:** Multivariable regression analysis for outcomes.

Multivariable linear regression analysis of Fertilization rate^ab^	Beta	CI for Beta	*p*-value
Abstinence period (days)	-1.42	-3.05, 0.20	0.087
Duration of infertility (Y)	-0.70	-1.20 ,-0.21	0.005
Progressive motility of sperm	1.48	0.40, 2.55	0.007
No. top-quality embryos transferred	6.33	1.71, 10.95	0.007
No. retrieved oocytes	-1.17	-1.75 ,-0.60	<0.001
**Multivariable linear regression analysis of Cleavage-stage embryo rate^ab^**	**Beta**	**CI for Beta**	***p*-value**
Early or secondary infertility	-3.52	-6.27 ,-0.77	0.012
Progressive motility of sperm	0.06	-0.002, 0.128	0.056
Sperm morphology	-0.64	-1.23 ,-0.041	0.036
No. oocytes aspirated	-0.35	-0.68 ,-0.06	0.017
**Multivariable logistic regression analysis of Implantation^ab^**	**OR**	**CI for OR**	**p-value**
Female age (Y)	0.96	0.92, 1.00	0.089
Etiology (combined vs. all)	2.05	1.30, 3.22	0.002
No. top-quality embryos transferred			
	1.92	1.20, 3.09	0.007
**Multivariable logistic regression analysis of Clinical pregnancy rate^ab^**	**OR**	**CI for OR**	***p*-value**
Female age (Y)	0.96	0.92, 1.00	0.072
Etiology (combined vs. all)	1.98	1.25, 3.14	0.003

a Regression modeling with a Backward approach.

b Variables entered for multivariable regression analysis: abstinence period, female age, male age, early or secondary infertility, previous ICSI cycles, ovarian stimulation protocol (agonist *vs*. antagonist), duration of infertility, semen volume, total sperm count, motility, progressive motility, sperm concentration, sperm morphology, number of aspirated oocytes, number of transferred embryos, etiology (combined *vs*. other, male factor *vs*. Other, unexplained *vs*. other). CI: confidence interval.

## DISCUSSION

To date, there is no clear, evidence-based recommendation for the sexual abstinence period before the ICSI treatment of infertile couples. We analyzed the outcome of 1003 fresh ICSI cycles to assess the importance of the sexual abstinence period and semen quality in the cycles. However, our findings demonstrated that some ICSI outcomes were associated with specific sperm parameters. The sexual abstinence period could affect some characteristics of the ejaculated semen.

It was reported in a systematic review that the effects of sexual abstinence on sperm parameters have shown that longer abstinence was associated with increased semen volume and total sperm count (Hanson *et al.*, 2018), but reports regarding the impact of abstinence on other semen parameters were contradictory and inconclusive (Hanson *et al.*, 2018). According to our findings, longer sexual abstinence results in higher total sperm count and increased semen volume, which is in accordance with previous reports (Raziel *et al.*, 2001; De Jonge *et al.*, 2004; Jurema *et al.*, 2005; Sobreiro *et al.*, 2005; Marshburn *et al.*, 2010; Sunanda *et al.*, 2014; Agarwal *et al.*, 2016; Welliver *et al.*, 2016; Hanson *et al.*, 2018). Besides, we noticed that the concentration, motility, and morphology of the sperm in the ejaculated semen were not affected by the duration of the sexual abstinence between 1 to 10 days. The longer sexual abstinence period resulted in the accumulation of the sperm in the epididymis, so, there was a high number of sperm in the ejaculated semen and also the semen volume was increased.

In this study, biochemical and clinical pregnancy and also cleavage-stage embryo rates were not significantly associated with sexual abstinence. Nevertheless, we found no significant association between sexual abstinence periods before ICSI and fertilization rate after the procedure. There are several studies with the same results in this regard (Colturato *et al.*, 2007; Lee *et al.*, 2015; 2018; Periyasamy *et al.*, 2017; Borges Jr *et al.*, 2019; Setti *et al.*, 2020). Lee *et al.* (2015) in a retrospective study have evaluated fertilization and pregnancy outcomes of 131 fresh ICSI cycles regarding different abstinence periods (2-4 days *vs*. 5-7 days). They found no significant difference between two groups of abstinence periods in fertilization rate, top-quality embryos on day 3, and clinical pregnancy rate (Lee *et al*., 2015). Another retrospective analysis of 449 fresh ICSI cycles has compared the clinical outcomes between different abstinence periods (2-7 days *vs*. ≥8 days) in groups of young maternal age (<38 years) and old maternal age (≥38 years). They demonstrated that fertilization rate, top-quality embryos on day 3, and pregnancy rate were not significantly different between 2-7 and ≥8 days of abstinence. They have also suggested that the sexual abstinence beyond the recommended abstinence period by WHO (2-7 days) was not associated with lower fertilization and pregnancy rates in ICSI cycles (Lee *et al*., 2018).

However, several of the preceding studies have indicated the sexual abstinence period was associated with embryo development and pregnancy outcomes after the ICSI procedure (Colturato *et al.*, 2007; Periyasamy *et al.*, 2017; Borges Jr *et al.*, 2019; Setti *et al.*, 2020). Setti *et al.* (2020) in a cohort study involving 427 oocyte recipient ICSI cycles showed that the abstinence period could affect embryo development and implantation. Moreover, in a prospective cohort study by Borges Jr *et al*. (2019), data from 818 ICSI patients were analyzed and the results showed that the ejaculatory abstinence period could influence implantation and pregnancy rates. They showed that 1-day of abstinence led to higher implantation and pregnancy rates compared to 2-4 days of abstinence (Borges Jr *et al.*, 2019). Colturato and colleagues, in a retrospective study of 445 ICSI cycles, compared pregnancy outcomes of ICSI cycles in different sexual abstinence periods (1, 2, 3, 4, 5, 6-10, and ≥11 days of abstinence). They showed that the highest pregnancy rate was achieved in 1-day abstinence before ICSI, and the lowest pregnancy rate was found in 5 days abstinence period. Also, five or more days of sexual abstinence period were associated with a decreased pregnancy rate. Also, they reported that a shorter sexual abstinence period was associated with higher embryo quality and an increased pregnancy rate (Colturato *et al.*, 2007). Periyasamy *et al.* (2017) analyzed outcomes of 1030 ICSI/IVF cycles according to the duration of the sexual abstinence period in two groups (2-7 days *vs*. >7 days of abstinence). They demonstrated that significantly higher implantation, pregnancy, and live birth rates were achieved in patients with 2-7 days of abstinence period, in comparison to >7 days of abstinence. Moreover, the highest pregnancy and live birth rates were noticed in a subgroup of patients with 2-4 days of abstinence period.

The results of our study showed that progressive motility of sperm could affect the fertilization rate in the ICSI cycle. Although there is no natural sperm selection in ICSI procedure and the best spermatozoa in the semen sample could be selected, Bartolacci *et al*. (2018) have reported that sperm motility was associated with fertilization rate but not with implantation in ICSI cycles. In accordance with our findings, they also have reported that the pregnancy outcomes of ICSI cycles did not associate with semen parameters (Bartolacci *et al.*, 2018). It has been proposed that disrupted spermatogenesis and DNA damages which lead to the development of an abnormal flagellum may affect the motility of spermatozoa (Waller *et al.*, 2019). Because of this, the significant positive association between progressive sperm motility and fertilization rate in the ICSI cycle might be due to possible disruption during spermatogenesis. There is very little evidence regarding the role of sperm morphology on embryo development (Danis & Samplaski, 2019), even so, our data demonstrated that the percentage of morphologically normal sperm was negatively associated with cleavage-stage embryo rate. This finding may indicate the importance role of sperm morphological abnormalities on the early stages of the embryo development.

The sexual abstinence period between 1 to 10 days has a significant association with the total sperm count and semen volume. The fertilization rate in the ICSI cycle is also associated with the progressive sperm motility in the preprocessing semen. The cleavage-stage embryo rate is negatively associated with the percentage of morphologically normal sperm in the preprocessing semen. Further, the pregnancy outcomes of the ICSI cycles are not affected by the abstinence period or the semen characteristics. We recommend future studies in a larger population to prove the impact of the abstinence period on the outcomes of ICSI cycles and also the prognostic value of semen parameters in the cycles.
